# Beyond the hours slept: inconsistent sleep routines threaten mental health in 100,000 UK Biobank participants

**DOI:** 10.1186/s12889-025-24794-7

**Published:** 2025-11-17

**Authors:** Max Moebus, Christian Holz

**Affiliations:** Department of Computer Science, ETH Zürich, Zurich, Switzerland

**Keywords:** Mental health, Mental disorders, Sleep, Routine sleep habits, Wearable sensors

## Abstract

**Background:**

Sleep duration has a well-established effect on mental health and well-being, with durations of 7 to 9 hours being the general recommendation. Here, we analyze the significance of sleep patterns and find that a consistent routine reduces the risk of developing mental disorders far more than simply ensuring a certain average sleep duration.

**Methods:**

We analyzed the sleep behavior of 100,000 adults for one week using motion data from wrist-worn devices. We modeled sleep behavior using multivariate generalized additive Cox proportional hazard models, incorporating a smooth 2D interaction effect of sleep duration and routine sleep hours. We calculated C-statistics and E-values to evaluate model performance and assess the robustness against hidden confounders. We also stratified analyses by age and gender.

**Results:**

Most participants slept for 7 to 9 hours as recommended, yet they consistently only slept during the same 4.8 hours each night. We found that an average sleep duration around 8 hours minimizes the risk of future mental disorders—but only if integrated into a rigorous sleep routine spanning at least the same 7 hours each night. Our study provides evidence that adopting such sleep behavior could reduce the population incidence rate of mental disorders by 23% (HR: 0.79, $p<0.0001$, for the average participant). The models showed a strong fit (C-statistics: 0.63), robustness to hidden confounders (E-value: 1.8), and stability under age- and gender-based stratification. We identified weekend behavior as a frequent reason for low sleep routines, with over 25% of the population disrupting their weekly sleep routine during weekend nights—raising the risk of future mental disorders by 10%.

**Conclusions:**

Our results suggest that maintaining a consistent sleep routine is more important for mental health than sleep duration alone. Socially disadvantaged groups, including low-income households and ethnic minorities, exhibited poorer sleep routines and thus higher mental disorder risks, underscoring existing social inequalities. Promoting regular sleep behavior may therefore have significant public health benefits.

**Supplementary Information:**

The online version contains supplementary material available at 10.1186/s12889-025-24794-7.

## Background

Mental disorders have profound implications for individuals and society at large. They encompass conditions such as depression, anxiety, and bipolar disorder. These conditions can hinder personal well-being, relationships, and workplace productivity, and place significant strain on healthcare systems [1–4]. The Broader societal and economic repercussions of untreated or inadequately managed mental health issues are vast and undeniable. Already in 2019, mental disorders were the leading cause of years lived with disability (one out of six years) at an estimated cost of one trillion USD per year to the global economy [5]. The recent COVID-19 pandemic further exacerbated these challenges, introducing an unprecedented combination of stressors including social isolation, health anxieties, and economic disruptions [6]. In 2020 alone, mental disorders are estimated to have increased by 26%–28% worldwide [7]. The surge in mental health concerns during the pandemic, but also as part of the post-COVID syndrome (long COVID) [8, 9], accentuates the pressing need for a better understanding of related risk factors.

Sleep has long been recognized for its role in emotional regulation, cognitive function, and overall health [10–12]. Sufficient sleep duration and its effect on mental well-being have been investigated in numerous studies [12–15]. There is overwhelming evidence that adequate sleep (e.g., durations of 7–9 hours [16]) reduces the risk of a Broad range of diseases including cardiovascular disease, type 2 diabetes, obesity, and many mental disorders such as depression [12, 17–19]. To aid with achieving sufficient sleep duration, various health organizations have recommended regular sleep habits, such as always going to bed at the same time [20]. Especially for children, for whom sufficient sleep is of particular importance [21, 22], regular sleep habits proved instrumental during the COVID pandemic when daily routines and structures such as school or sports were removed [23] as already indicated by earlier works [24–26]. For adults, there are strong indications that regular bed and wake times, little variation in sleep duration, and high sleep efficiency should all promote mental health [27–35]—all of which points to the importance of sleeping *routinely* for the same hours each night [36].

However, the importance of routine sleep (sleeping for the same hours each night) even when sufficient sleep duration is generally achieved remains unclear. In a society where erratic schedules [37, 38] and, similarly, mental disorders are becoming commonplace [5], the potential link between sleep routines and the risk of developing mental disorders is of paramount importance.

In this paper, we analyze the effect of routine sleep on mental disorders challenging current recommendations for healthy sleep habits, comparing the effect of routine to that of mere duration of sleep. We introduce the metric of ‘routine sleep hours’ counting the number of hours that participants are routinely asleep, which combines the notions of sufficient sleep duration, consistent sleep duration, sleep continuity, and regular sleep onset times. For 100,000 participants of the UK Biobank (UKB) we passively assess sleep routine using wrist-worn accelerometers during a one-week sleep behavior study. Through multivariate generalized additive Cox proportional hazard models, we calculate the optimal sleep routine and its effect on the onset of mental disorders. We construct a smooth 2D interaction effect between mere sleep duration and routine sleep hours—our metric of sleep routine. In contrast to related works that investigated the relation between sleep duration and mental health using 1D (linear or non-linear) effects, our 2D effect adequately captures the complexity of sufficient sleep duration being integrated within a rigorous routine. Additionally, we investigate daily routines during weekends, which commonly disrupt sleep patterns.

## Methods

### Study population

Our analysis is based on 103,661 participants of the UKB who participated in a one-week substudy wearing an accelerometer capturing wrist motion (sleep behavior study). For 98,470 participants pre-computed activity profiles were available according to previously validated models [39]. For 5,191 participants, activity profiles were not computed due to low compliance [39]. 56.3% of participants were female and the median age at the time of the sleep behavior study was 63.5 years with an inter-quartile range (IQR) of 12.3 years. More details are provided in Table 1.

**Table 1:** Comparison of routine sleep hours (RSH), average sleep duration (ASD), non-routine sleep hours (NRSH), and average awake duration (AAD) between different subgroups of participants. For RSH, we show the mean (RSH$_m$) and median (RSH$_{1/2}$). The *p*-value corresponding to Wilcoxon signed rank tests (two-sided) for mean comparison of RSH for each subgroup against all other participants is given in the *p*-val column. The *MD*$_f$ and *MD*$_p$ columns give the percentage of people who developed a mental disorder after and prior to the sleep behavior study, respectively. For the percentage in the *MD*$_f$ column, participants who had developed a mental disorder prior to the sleep behavior study were excluded. The size of each subgroup is given in column *n*. Where applicable, the unit is given in the first row for each criterion. For each criterion separately, participants who did not provide information or who responded ‘Do not know‘ or ‘Prefer not to answer’ were removed from the table. Thus, for some criteria, the *n* column does not sum to 98,470

Criterion	Condition	RSH$_m$	RSH$_{1/2}$	ASD	NRSH	AAD	*p*-val	MD$_f$	MD$_p$	n
	All	4.77	5	8.90	4.13	15.10		3.4%	15.2%	98470
Sex	Female	4.97	5	8.94	3.97	15.06	<0.001	3.9%	17.0%	55447
Sex	Male	4.51	5	8.85	4.34	15.15	<0.001	2.8%	10.9%	43023
Age	< 56.2 (y)	4.74	5	8.76	4.02	15.24	<0.001	3.5%	16.1%	24205
Age	56.2 to 68.6	4.82	5	8.92	4.10	15.08	<0.001	3.1%	14.7%	49304
Age	>68.6	4.69	5	8.99	4.30	15.01	<0.001	3.8%	11.9%	24931
Smoking	Never	4.89	5	8.89	4.00	15.11	<0.001	3.0%	13.3%	56065
Smoking	Previous	4.67	5	8.91	4.24	15.09	<0.001	3.8%	15.1%	35334
Smoking	Current	4.24	5	8.95	4.71	15.05	<0.001	4.9%	19.3%	6807
Alcohol	Never	4.52	5	8.92	4.39	15.08	<0.001	4.1%	17.1%	2846
Alcohol	Previous	4.39	5	8.98	4.58	15.02	<0.001	5.7%	23.7%	2712
Alcohol	Current	4.79	5	8.90	4.11	15.10	<0.001	3.3%	14.0%	92821
Ethnicity	White	4.79	5	8.91	4.11	15.09	<0.001	3.4%	14.4%	95079
Ethnicity	Asian	4.15	4	8.74	4.59	15.26	<0.001	2.6%	10.5%	1307
Ethnicity	Black	3.55	4	8.51	4.96	15.49	<0.001	4.2%	10.6%	1006
Ethnicity	Other	4.24	5	8.78	4.54	15.22	<0.001	4.2%	12.9%	1078
Income	< 18k (£/y)	4.53	5	9.09	4.55	14.91	<0.001	5.1%	20.5%	13033
Income	18k to 30k	4.72	5	8.98	4.26	15.02	0.005	3.8%	15.5%	21374
Income	31k to 52k	4.79	5	8.87	4.08	15.13	0.057	3.2%	14.2%	25322
Income	52k to 100k	4.87	5	8.77	3.91	15.23	<0.001	2.4%	11.6%	22138
Income	>100k	4.84	5	8.67	3.83	15.33	0.078	1.7%	8.4%	6428
Night shifts	Always	3.25	4	8.81	5.55	15.19	<0.001	2.5%	16.1%	971
Night shifts	Sometimes	4.14	4	8.82	4.68	15.18	<0.001	3.5%	13.1%	2272
Night shifts	Usually	3.58	4	8.94	5.35	15.06	<0.001	3.9%	17.6%	618
Night shifts	Never/rarely	4.50	5	8.85	4.35	15.15	<0.001	3.9%	16.5%	4211
Work week	< 28 (h)	4.97	5	8.93	3.95	15.07	<0.001	3.3%	15.4%	15382
Work week	28 to 42	4.75	5	8.84	4.09	15.16	0.229	3.2%	14.4%	28230
Work week	>42	4.54	5	8.65	4.12	15.35	<0.001	2.5%	10.7%	13944

### Measuring sleep routine via routine sleep hours

**Fig. 1:**
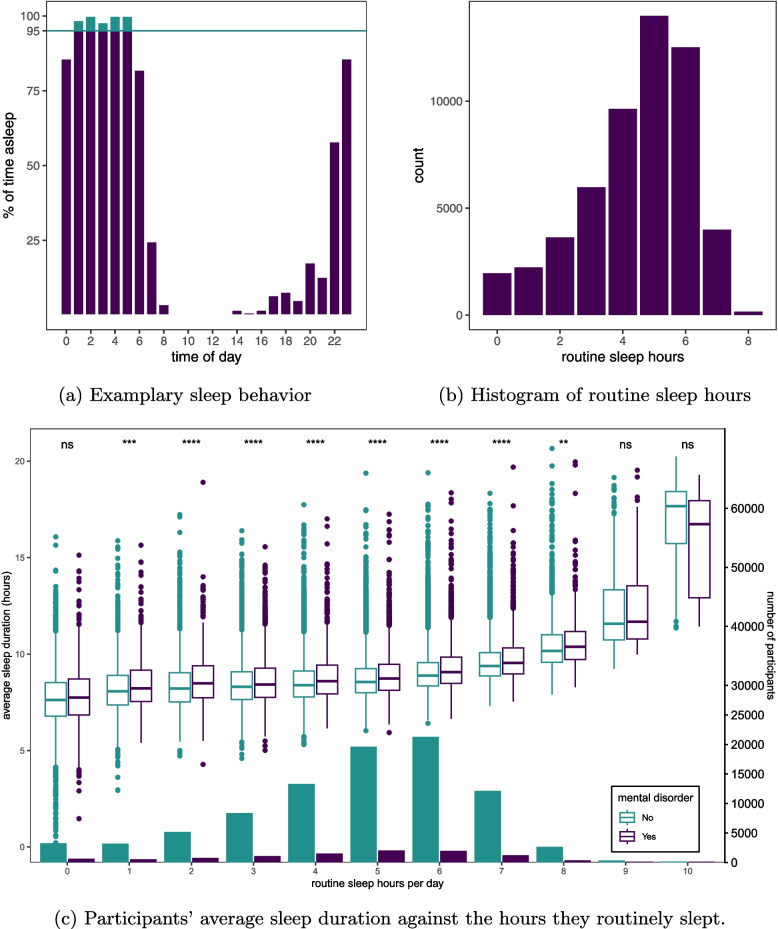
Sleep behavior of participants who meet the commonly recommended average sleep duration of 7–9 hours per night. **a** Examplary sleep behavior of a participant maintaining a sleep routine that covered the same 5 hours each night at an average sleep duration of 8.9 hours. **b** Histogram of routine sleep hours for participants who slept 7–9 hours per night. **c** Participants’ average sleep duration against the hours they routinely slept during the sleep behavior study, split according to prior diagnosis of a mental disorder. Differences in sleep duration per level of routine sleep hours depending on prior diagnosis: $$^{****} p \le 0.0001$$, $$^{***} p \le 0.001$$, $$^{**} p \le 0.01$$, *ns* $$p> 0.05$$ according to two-sided Wilcoxon signed rank tests. The secondary axis on the right is for the bottom histogram for each group

We assess sleep routine based on the one-week sleep behavior study of the UKB. Using previously verified activity recognition models [39], we assess for what percentage of the time participants were asleep during 24 one-hour Brackets for each of the 7days. For each one-hour Bracket when participants were asleep for at least 95% of the time, we count one hour of sleep routine. To determine the optimal threshold that defines a window as a routine sleep hour, we computed the average number of routine sleep hours across the entire study population for possible threshold between 1% and 99% (with 1% increments). The threshold of 95% represents the highest threshold before the average number of routine sleep hours in the entire study population starts to drop drastically towards 0. Thus, thresholds beyond 95% appear to be unnaturally high or impossible to achieve due to potentially inaccurate sleep classification. An example sleep behavior profile is displayed in Fig. A1 which corresponds to 5 routine sleep hours. Fig. 1a shows the sleep behavior of an average participant who maintains a sleep routine covering the same 5 hours each day. A detailed visualization of the logic of routine sleep hours is shown in Fig. A1.

### ICD-10 codes used to identify mental disorders

We extracted the diagnosis of mental disorders from clinical and primary care records using the ICD-10 Codes F20–F69. There was no participant diagnosed with ICD-10 Codes F60–F69, who had not been diagnosed with a different mental disorder. The censoring date for participants registered with NHS England was in October 2022, in August 2022 for Scottish participants, and in May 2022 for Welsh participants [40].

### Statistical analysis

For an initial analysis, we used two-sided Wilcoxon signed rank tests to test for differences in routine sleep hours between subgroups of the study population in Table 1. To predict the risk of developing mental disorders in the future we used multivariate generalized additive Cox proportional hazard models. To this end, we used the ‘MGCV‘ package [41] to calculate non-linear and 2D effects. For non-linear effects, we fitted thin plate regression splines as generally recommended [42]. Motivated by related work [33], we controlled for age, sex, body mass index (BMI), smoking status, alcohol consumption, household income, time spent in a state of high physical activity, fruit and vegetable consumption, smartphone use, computer use, and TV use. While the time spent in a state of high physical activity is passively assessed during the week-long sleep behavior study [39], information about participants’ household income, smoking status, alcohol consumption, fruit and vegetable consumption, smartphone use, and computer use was assessed during a questionnaire upon recruitment into the UK Biobank between 2006 and 2010. After including all of the aforementioned control variables in the multivariate generalized additive Cox proportional hazard model, we iteratively removed the effect with the highest *p*-value until all effects were significant with a *p*-value $\le$ 0.1 [43, 44]. This removed alcohol consumption and smartphone use from the model.

### Sensitivity analysis and model validation

We extensively tested the validity, sensitivity, and robustness of our multivariate generalized additive Cox proportional hazard model following established guidelines for Cox proportional Hazards models [45–47]. Via cross-validation, we tested the performance and generalizability of our model comparing predicted survival to Kaplan-Meier curves visually and based on Uno’s and Harrel’s *C*-statistic [48, 49]. During 10-fold cross-validation, we computed *C*-statistics for the training as well as the test set, which allowed us to assess the model’s optimism and identify any structural overfitting. To assess the sensitivity of our model towards hidden confounders, we calculated the E-value [50] for the 2D sleep routine effect which measures the necessary strength of the relationships between a hidden confounder and, both, the risk of developing a mental disorder as well as sleep behavior to explain away the measured effect.

We further assessed the robustness of the model fit by constructing the multivariate generalized additive Cox proportional hazard model for four subgroups of the study population separately. We fitted the multivariate generalized additive Cox proportional hazard model once for female, male participants, younger, and older participants separately (Fig. A2a–e). The 2D sleep routine effect remains constant and results in nearly the same optimal sleep routine demonstrating general robustness towards sex-based stratification. The shape of the 2D sleep routine effect also remained constant when removing any of the aforementioned control variables—again demonstrating a robust model fit.

To rule out reverse causality (at least partially) and further assess the robustness of our model, we calculated the model ignoring any diagnosis within 2 years of the sleep behavior study. However, mental disorders are commonly diagnosed very late or not at all [51]. Thereby, reverse causality remains a limitation, especially given the oftentimes bi-directional relationship between sleep and mental disorders [52].

We carefully evaluated the proportional hazards assumption for the 2D sleep routine effect by visually inspecting score residuals [41, 53].

## Results

We first analyze sleep routines via our novel metric of ‘routine sleep hours’ and mental disorder incidence across subgroups of the population. Subsequently, we assess how sleep routines might be affected through mental disorders. Finally, we investigate the effects of specific sleep routines on mental disorder onset using multivariate generalized additive Cox proportional hazard models during an average follow-up of 8 years.

### Sleep routine and mental disorder incidence across subgroups

Sleep behavior, and sleep routine, vary significantly across different subgroups of the population based on sex, age, ethnicity, household income, alcohol consumption, smoking status, or work environment (Table 1). The average participant slept for 8.9 hours per day and maintained a sleep routine covering the same 4.8 hours each day. Subgroups of the population such as male participants (4.5 h), current smokers (4.2 h), participants of black ethnicity (3.6 h), participants with an annual household income of less than 18.000 GBP (4.5 h), participants whose job involves night shifts, or participants with a work week of 42 hours or longer (4.5 h) sleep significantly less routinely than the rest of the population. Overall, 15.2% of all participants had been diagnosed with a mental disorder before the start of the sleep behavior study. For current smokers (19.3%), participants who used to consume alcohol but not anymore (23.7%), and participants with an annual household income below 18.000 GBP (20.5%) this proportion was highest. Out of the participants who did not develop a mental disorder prior to the sleep behavior study, 3.4% developed a mental disorder during an average follow-up period of 8.0 years. The incidence rate of mental disorders was highest for female participants (3.9%), current smokers (4.9%), participants who used to drink alcohol but not anymore (5.7%), participants of black ethnicity (4.2%), those categorized under ‘Other’ ethnicities, (4.2%), and participants with an annual household income below 18.000 GBP (5.1%).

With participants of black ethnicity, those categorized under ‘Other’ ethnicities, and those with a household income below 18.000 GBP, ethnic minorities and socially disadvantaged groups show, both, less regular sleep patterns and a high incidence of mental disorders.

### Sleep routine is more than just sleeping enough

Participants who slept on average 7 to 9 hours per day during the sleep behavior study (55% of participants) achieved a sleep routine that covers on average the same 4.5 hours each day with a mode of 5 hours (Fig. 1b). Only 7.8% achieved a sleep routine that covered more than the same 6 hours each day. Figure 1c shows the relationship between average sleep duration and number of routine sleep hours across all participants with an overall correlation of 0.41 (Spearman, $p<$ 0.0001). Most participants who had been diagnosed with a mental disorder prior to the sleep behavior study maintained a sleep routine covering the same 5 hours each day. For participants who had not been diagnosed with a mental disorder, the most common sleep routine covered the same 6 hours per day. For sleep routines covering the same 1–8 hours per day, participants who had been diagnosed with a mental disorder before the sleep behavior study slept on average significantly longer ($p<$ 0.01) than participants who had not been diagnosed with a mental disorder—at each level of routine sleep hours.

### Mental disorders hazard model

To assess if less routine sleep hours also increase the risk of developing mental disorders in the future, we constructed multivariate generalized additive Cox proportional hazard models [41]. Our model calculates the 2D sleep routine effect: the smooth non-linear 2D effect of routine sleep hours and average sleep duration. In line with related work, we controlled for age, sex, body mass index (BMI), smoking status, alcohol consumption, household income, time spent in a state of high physical activity (passively assessed during the sleep behavior study), fruit and vegetable consumption, smartphone use, computer use, and TV use based on related work [33].

**Fig. 2:**
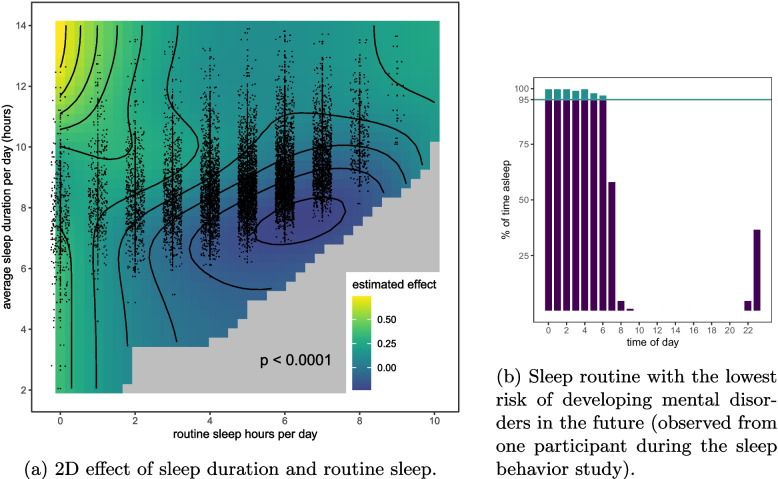
**a** The calculated 2D effect of average sleep duration and routine sleep hours on the hazard function of developing a mental disorder in the future (positive values increase the risk). Observed data points are presented as dots with jitter in the horizontal axis. Areas too far away from any data point are gray. **b** The observed sleep routine with the lowest risk of developing a mental disorder is 6 routine sleep hours at an average sleep duration of 6.8 hours

**Fig. 3:**
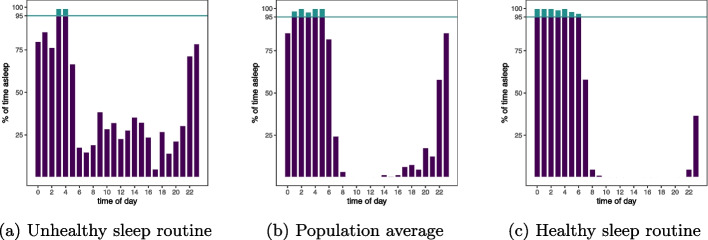
Three participants’ sleep routine. **a** A participant with a high risk of developing mental disorders due to their sleep routine (2 routine sleep hours and average sleep duration of more than 10 hours). **b** A participant representative of the population average (5 routine sleep hours and 8.9 hours average sleep duration). **c** The observed sleep routine with the lowest associated risk of developing mental disorders (7 routine sleep hours and 8 hours average sleep duration)

Figure 2 displays the 2D sleep routine effect ($p < 0.0001$, positive values correspond to an increased risk of developing mental disorders). Calculated effects for the control variables are illustrated in Fig. A3. We find that 7 routine sleep hours at an average sleep duration of 8 hours per day minimizes the risk of developing mental disorders in the future. Figure 3 shows three example sleep routines: the routine for the average participant and a routine with a particularly low and particularly high risk of mental disorders. Switching from the sleep behavior of the average participant (5 routine sleep hours and 8.9 average sleep duration) to what we calculate as the optimal sleep behavior corresponds to a Hazard ratio (HR) of 0.79 ($p < 0.0001$).

### Sensitivity Analysis

To assess the robustness of our calculated 2D sleep routine effect, we conducted a thorough sensitivity analysis. The calculated Hazard ratio of 0.79 corresponds to an E-value of 1.83 indicating strong robustness against hidden confounders [50]. As mentioned above, we have included a wide range of control variables to rule out such hidden confounders [33]. The constant shape of the 2D sleep routine effect when removing subsets of the aforementioned control variables from the model, and during age-/gender-based stratification (Fig. A2b–e) is another sign of robustness. The 2D sleep routine effect also remained constant when ignoring any diagnosis that occurred within 2 years after the sleep behavior study suggesting a long-term effect and no reverse causality (Fig. A2f).

However, there is likely slight variation in the optimal sleep routine between individuals. Figure A2a–e all show that less than 8 hours average sleep duration might still be close to optimal as long as they are integrated into a rigorous routine: e.g., 7 hours average sleep duration with at least 6 hours of routine sleep. Across 10 splits, we evaluated our model by randomly leaving out 10% of the data as an unseen test split. On the training splits, the model achieved an average Harrel’s and Uno’s *C*-statistic of 0.634 [48, 49]. On the test splits, the model achieved an average *C*-statistic of 0.625. As highlighted in Fig. A4, such C-statistics demonstrate good model fit and no excessive optimism.

### Potential to reduce mental disorder incidence

**Fig. 4:**
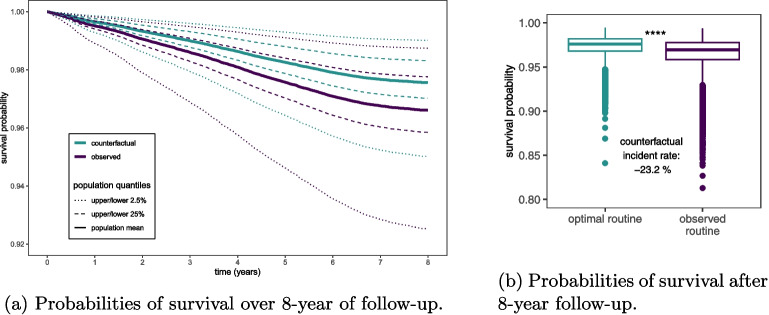
The probabilities of survival (i.e., the probability of not developing a mental disorder) per year are aggregated for all participants. We compare the calculated probabilities of not developing a mental disorder for all participants if they behaved as observed, and for the—according to our calculations—optimal but counterfactual case where all participants sleep for an average of 8 hours per day and for 7 hours routinely each day. **a** depicts the development of the whole 8-year follow-up. **b** shows the significant difference in survival probability after the 8-year follow-up ($$^{****}: p$$-val < 0.0001, two-sided Wilcoxon signed rank test)

Our study provides evidence that incorporating sufficient sleep duration into a rigorous sleep routine could reduce the population incidence rate of mental disorders by 23%. Based on our multivariate generalized additive Cox proportional hazard model, we calculated an optimal sleep routine: a sleep routine that covers at least the same 7 hours each day at an average sleep duration of 8 hours. Figure 4 displays the probabilities of not developing a mental disorder across an 8-year follow-up aggregated over all participants if everyone had adopted what we calculated as the optimal sleep routine: maintaining a sleep routine covering the same 7 hours each day at an average sleep duration of 8 hours (counterfactual). We simulate this counterfactual scenario by simply replacing the observed sleep routines, with what we calculate as the globally optimal sleep routine leaving all other variables unchanged [54]. We calculate that if everyone had adopted an average sleep duration of 7 to 9 hours, without paying attention to sleep routine, we calculate only a 3% reduction in mental disorder incidence at a population level.

## Changes in sleep routine on the weekend

**Fig. 5:**
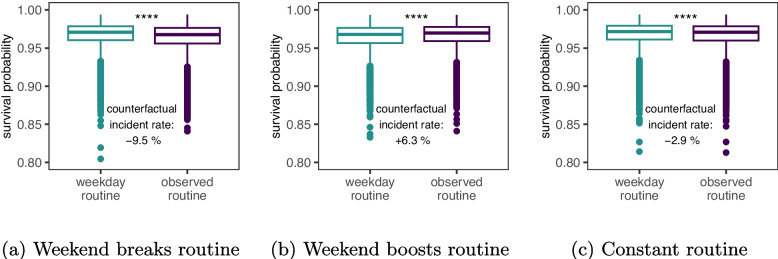
Mental disorder incidence rate changes due to weekend sleep routine. **a** 26.6% of participants Broke their sleep routine on the weekend. Keeping the same sleep pattern on weekends would have reduced the incidence rate by 9.5%. **b** 17.6% of participants improved their sleep routine on the weekend. Keeping the same sleep pattern on weekends would have increased the incidence rate by 6.3%. **c** 55.8% of participants maintained the same number of sleep routine hours on the weekend. But, on average, they increased their sleep duration on the weekend. Keeping the same sleep pattern on weekends would have reduced the incidence rate by 2.9%. The survival probabilities in **a**–**c**, if participants had continued their sleep routine on the weekend (counterfactual), are calculated using the multivariate generalized additive Cox proportional hazard models. Changes in weekend behavior lead to significantly different survival probabilities ($$^{****}:p$$-val < 0.0001) according to two-sided Wilcoxon signed rank tests

Over the weekend, parts of the population change their sleep routine increasing their risk of mental disorders. When re-calculating the number of ‘routine sleep hours’ only during weekdays, we find that 26.6% of participants broke their sleep routine on the weekend decreases their achieved number of ‘routine’ sleep hours. We calculate that such disruptions in sleep routine increase their risk of developing a mental disorder by 10% (Fig. 5a). On the other hand, 17.6% of participants increased their routine sleep hours over the weekend, which we calculate decreased their risk of developing a mental disorder by 6% (Fig. 5b). More than 55% of the population maintained the same number of routine sleep hours on the weekend. However, on average they slightly increased their sleep duration, which we calculate to increase their risk of developing a mental disorder by 3% (Fig. 5c).

## Discussion

We demonstrated the importance of a rigorous sleep routine that accommodates sufficient sleep duration to reduce the risk of developing an ever more common medical condition: mental disorders. We calculate that adopting a sleep routine covering the same 7 hours alone has the potential to reduce mental disorder incidence by 23%. On average, individuals who achieve an average sleep duration of 7–9 hours—as commonly recommended [55]—only manage to maintain a sleep routine of less than the same 4.5 hours each night. This discrepancy indicates the need for large parts of the population to rethink their daily routines to foster adequate sleep routines. Health organizations might promote this change in sleep behavior by updating their recommendations to stress the importance of sleep routines. Ideally, one sleeps at the same time each night covering one’s need for sleep each night.

Our analysis also surfaces that socially disadvantaged groups, such as ethnic minorities or low-income households, exhibit significantly fewer routine sleep hours. The incidence of mental disorders is particularly high in these groups, which highlights a known social inequality in health as also observed for various other diseases [56–59]. The significant difference in sleep routine of socially disadvantaged groups as observed in our study offers a possible explanation for this known social inequality related to mental disorders [60].

Using our metric of ‘routine sleep hours’, we unify multiple ideas from a large body of literature on healthy sleep: sufficient sleep duration, consistent sleep durations, sleep continuity, and regular sleep onset times. Although we argue against sleep duration as the main measure of identifying healthy sleep behavior, our work highly agrees with existing works on the importance of sleep [12–15]. Healthy sleep is well recognized to decrease the likelihood of mental disorders [12–15] and conditions like cardiovascular diseases and type 2 diabetes [12, 17–19], as also supported by extensive studies using the UK Biobank [30]. Our study highlights an aspect of sleep behavior that is somewhat neglected in previous work as well as in recommendations by health organizations: integrating sufficient sleep duration into a rigorous sleep *routine*. Our results provide a unifying view on an extensive body of work that showed the importance of regular sleep and wake times, reduced variability in sleep duration, and sleep efficiency for adults’ overall and mental health [13, 27–32, 34]. Similarly, our results reinforce various works that showed the importance of regular sleep habits for children’s overall health and development [24–26]. A rigorous sleep routine would result in minimal variability in both sleep duration and bedtime, which is in line with findings from related studies [13, 30–32, 34, 36]. Our metric of sleep routine goes further than what has been considered in previous works. Metrics such as the Sleep Regularity Index (SRI) [35], while related and likely correlated, consider only day-to-day changes in sleep behavior and ignore longer term trends. Thus, falling short of assessing actual routine behavior. Ideally, such a sleep routine aligns with individuals’ circadian rhythm [61].

In contrast to related works, our analysis accounts for a smooth 2D interaction effect between passively assessed sleep duration and routine sleep hours. As opposed to 1D (linear or non-linear) effects, the 2D effect captures the complexity of sufficient sleep duration integrated within a rigorous routine. We are thus able to draw a much more nuanced conclusion than previous works: an average sleep duration of 8 hours integrated into a rigorous sleep routine covering at least the same 7 routine sleep hours significantly reduces the risk of future mental disorders. The 2D effect also shows that also an average sleep duration of 7 hours might be associated with low mental disease risk, if integrated into a routine that spans at least 6 routine sleep hours. It is the added complexity of our model that allows to investigate the simultaneous importance of sleep duration and routine sleep hours.

Our analysis also underscores the bidirectional relationship between sleep and mental disorders. Similar to related studies [30, 62], we find that individuals affected by mental disorders sleep significantly differently to healthy individuals. Past research found that individuals affected by mental disorders often develop sleep apnea [52], insomnia [63], or hypersomnia [64]. However, these conditions were also identified as risk factors for developing mental disorders. Thus, it is not surprising to find a similar relationship between mental disorders and sleep routine—especially since insomnia, hypersomnia, and sleep apnea seem likely to affect sleep routine. To ensure our model does indeed provide evidence of a bidirectional relationship and does not simply capture reverse causality, we also evaluated our model when excluding any diagnosis within 2 years after the sleep behavior study [54].

Our metric of sleep routine—routine sleep hours—is affected by participants’ sleep duration and the general regularity of their sleep. While only excessive daytime napping would affect our sleep routine metric, participants’ sleep continuity is likely to have an effect. By counting all 1-hour Brackets when participants were asleep for at least 95% of the time, we allow for 3 minutes of wakefulness on average in each of the 24 1-hour Brackets, which accumulates to 21 minutes across the 7 days of the sleep behavior study. If participants woke up during the same 1-hour Bracket on multiple days during the 1-week sleep behavior study, this might reduce the number of routine sleep hours. Since we base our sleep routine metric on 24 fixed 1-hour brackets (from midnight to 1 am, from 1 am to 2 am, $\ldots$, from 11 pm to midnight), it might underestimate routine sleep if participants do not fall asleep at a full hour and wake up exactly at a full hour. Thus, it is likely that the optimal number of routine sleep hours to minimize the risk of developing mental disorders lies somewhat higher—closer to our calculated optimal sleep duration of roughly 8 hours.

Our study provides evidence for future sleep recommendations to highlight more clearly the importance of incorporating sufficient sleep duration into a rigorous sleep routine, helping realize the calculated potential reduction in population incidence of mental disorders of up to 23%. We calculate that at least 7 routine sleep hours at an average sleep duration of 8 hours minimize the risk of developing mental disorders in the future. However, there will be some variability in this optimum between different individuals as also visible in Fig. A2a—e. While ever more erratic schedules [37, 38] might contribute to less consistent sleep routines, our analysis highlights that less sleep overall might be more beneficial for a large proportion of the population—as long as it is integrated into a healthy sleep routine.

Participants are particularly likely to change their daily routines and, subsequently, also their sleep routine on the weekend. We find that 27% of participants disrupt their sleep routine on the weekend. In contrast, 18% of participants used the weekend to increase their number of routine sleep hours. Participants who kept their number of sleep routine hours constant over the weekend (56%), on average increased their sleep duration on the weekend. Participants who used the weekend to increase their number of routine sleep hours decreased their risk of developing mental disorders by 6%, while participants who Broke their sleep routine on the weekend increased their risk of developing mental disorders by 10%. This stresses the potential of the weekend to recover from potentially disrupted patterns during the week, as well as the danger of breaking any routine built up during the week.

The sensitivity analysis of our multivariate generalized additive Cox proportional hazard model indicates a robust 2D sleep routine effect. Predicted and observed survival curves show good agreement in Fig. A4, as also confirmed by Harrel’s and Uno’s C-statistics of 0.634—which lies well within the acceptable range of 0.6–0.75 for biomedical survival analysis [65, 66]. For the average participant, the 2D sleep routine effect implies a Hazard ratio of 0.79 ($p<0.0001$) decreasing the risk of developing mental disorders when adopting the optimal sleep routine of at least 7 routine sleep hours at an average sleep duration of 8 hours. This corresponds to an E-value of 1.83, indicating the necessary strength of a hidden confounder on both sleep behavior and mental disorder risk to explain the 2D sleep routine effect [50], which exceeds similar survival studies also conducted on large-scale observational studies such as the UKB [54]. Since we control for established risk factors [33], such a hidden confounder is particularly unlikely. Age- and sex-based stratification indicate robust behavior of the 2D sleep routine effect across different subgroups in Fig. A2a–e. Figure A2f further shows the robustness of the 2D sleep routine effect when excluding any diagnosis within 2 years after the sleep behavior study. This rules out reverse causality in case some mental disorders were simply not diagnosed at the time of the sleep behavior study but would have affected sleep behavior as shown in Fig. 1c and also indicates that the 2D sleep routine effect remains constant over time. We assessed the proportional hazards assumption for the 2D sleep routine effect by inspecting score residuals [41, 53] and found evidence to reject it ($p<0.05$), which indicates that the main model assumption of our multivariate generalized additive Cox proportional hazard model is valid and that there is no change in the 2D sleep routine effect over time.

Our study has several limitations. First, our calculations might actually underestimate the prevalence of mental disorders. Despite its large study size, the UKB is subject to a well-documented healthy volunteer bias and thus not representative of its sampling population [67, 68]. Generally, many mental disorders go undetected [51, 69]. Our calculated optimal sleep routine might thus not be optimal for the whole population, which is amplified by the high average age of the UKB cohort (63.5 years with an IQR of 12.3 years). Another limitation is the four to eight years between recruitment (2006–2010), when information such as household income was reported, and the sleep behavior study (2014) of the UKB. It is unlikely that the information listed in Table 1 was up-to-date for all participants at the beginning of the sleep behavior study. It is also unknown how well the 1-week sleep behavior study represents participants’ true daily and sleep routines. Most likely, for a single participant, the 1-week sleep behavior study is a noisy observation of their overall routine. The noise level is amplified potentially by inaccurate sleep detection, which is still not perfect based on wrist-worn accelerometers [70, 71]. The true importance of sleep routine might thus be understated in our analysis and would require more long-term modeling of participants’ routines.

## Conclusions

Sleep routine, assessed via our novel metric of ‘routine sleep hours,’ shows a strong, likely bi-directional, relationship to mental disorders: poor routine increases the risk of mental disorders, while mental illness likely also impairs routine. While optimal sleep behavior remains individual, we estimate that adopting a sleep routine that covers at least the same 7 hours each night at an average sleep duration of 8 hours could reduce the population-wide incidence of mental disorders by 23%. We find weekends to be a common reason for disrupted sleep routines. Moreover, our analysis highlights a critical social dimension: individuals from low-income households and ethnic minority groups are disproportionately affected by poor sleep routines and higher mental health risks.

## Supplementary Information

Supplementary material. Fig. A1: Derivation of the number of routine sleep hours for an exemplary participant. Fig. A2: Stratification of the 2D sleep routine effect. Fig. A3: Effects of the Control Variables. Fig. A4: Kaplan Meier survival curves.

## Data Availability

The data used in our manuscript are available from the UKB [72] at the discretion of the UKB. Bona-fide researchers can apply for access to the UKB (https://www.ukbiobank.ac.uk/enable-your-research/apply-for-access). The accompanying code is available to the reviewers via PolyBox and will be publicly available via GitHub upon publication.

## References

[1] Alonso J, Angermeyer M, Bernert S, Bruffaerts R, Brugha T, Bryson H, et al. Disability and quality of life impact of mental disorders in Europe: results from the European Study of the Epidemiology of Mental Disorders (ESEMeD) project. Acta Psychiatr Scand. 2004;109:38–46. 15128386 10.1111/j.1600-0047.2004.00329.x

[2] Docrat S, Besada D, Cleary S, Daviaud E, Lund C. Mental health system costs, resources and constraints in South Africa: a national survey. Health Policy Plan. 2019;34(9):706–19. 31544948 10.1093/heapol/czz085 PMC6880339

[3] Braithwaite S, Holt-Lunstad J. Romantic relationships and mental health. Curr Opin Psychol. 2017;13:120–5. 28813281 10.1016/j.copsyc.2016.04.001

[4] Bubonya M, Cobb-Clark DA, Wooden M. Mental health and productivity at work: does what you do matter? Labour Econ. 2017;46:150–65.

[5] WHO. World mental health report: transforming mental health for all. World Health Organization; 2022.

[6] Hossain MM, Tasnim S, Sultana A, Faizah F, Mazumder H, Zou L, et al. Epidemiology of mental health problems in COVID-19: a review. F1000Res. 2020. 10.12688/f1000research.24457.1. PMC7549174 33093946

[7] Organization WH, et al. Mental health and COVID-19: early evidence of the pandemic’s impact. World Health Organization; 2022.

[8] Renaud-Charest O, Lui LM, Eskander S, Ceban F, Ho R, Di Vincenzo JD, et al. Onset and frequency of depression in post-COVID-19 syndrome: A systematic review. J Psychiatr Res. 2021;144:129–37. 34619491 10.1016/j.jpsychires.2021.09.054 PMC8482840

[9] Anaya JM, Rojas M, Salinas ML, Rodríguez Y, Roa G, Lozano M, et al. Post-COVID syndrome. A case series and comprehensive review. Autoimmun Rev. 2021;20(11):102947. 34509649 10.1016/j.autrev.2021.102947 PMC8428988

[10] Hublin C, Partinen M, Koskenvuo M, Kaprio J. Sleep and mortality: a population-based 22-year follow-up study. Sleep. 2007;30(10):1245–53. 17969458 10.1093/sleep/30.10.1245 PMC2266277

[11] Milojevich HM, Lukowski AF. Sleep and mental health in undergraduate students with generally healthy sleep habits. PLoS ONE. 2016;11(6):e0156372. 27280714 10.1371/journal.pone.0156372 PMC4900547

[12] Palmer CA, Alfano CA. Sleep and emotion regulation: an organizing, integrative review. Sleep Med Rev. 2017;31:6–16. 26899742 10.1016/j.smrv.2015.12.006

[13] Lemola S, Ledermann T, Friedman EM. Variability of sleep duration is related to subjective sleep quality and subjective well-being: an actigraphy study. PLoS ONE. 2013;8(8):e71292. 23967186 10.1371/journal.pone.0071292 PMC3743871

[14] Kahn M, Sheppes G, Sadeh A. Sleep and emotions: bidirectional links and underlying mechanisms. Int J Psychophysiol. 2013;89(2):218–28. 23711996 10.1016/j.ijpsycho.2013.05.010

[15] Walker MP, van Der Helm E. Overnight therapy? The role of sleep in emotional brain processing. Psychol Bull. 2009;135(5):731. 19702380 10.1037/a0016570 PMC2890316

[16] Watson N, Badr M, Belenky G, Bliwise D, Buxton O, Buysse D, et al. Consensus Conference Panel. Joint consensus statement of the American Academy of Sleep Medicine and Sleep Research Society on the recommended amount of sleep for a healthy adult: methodology and discussion. Sleep. 2015;38(8):1161–83. 10.5665/sleep.4886 PMC4507722 26194576

[17] Wolk R, Gami AS, Garcia-Touchard A, Somers VK. Sleep and cardiovascular disease. Curr Probl Cardiol. 2005;30(12):625–62. 16301095 10.1016/j.cpcardiol.2005.07.002

[18] Ogilvie RP, Patel SR. The epidemiology of sleep and diabetes. Curr Diabetes Rep. 2018;18:1–11. 10.1007/s11892-018-1055-8 PMC6437687 30120578

[19] Beccuti G, Pannain S. Sleep and obesity. Curr Opin Clin Nutr Metab Care. 2011;14(4):402. 21659802 10.1097/MCO.0b013e3283479109 PMC3632337

[20] Irish LA, Kline CE, Gunn HE, Buysse DJ, Hall MH. The role of sleep hygiene in promoting public health: a review of empirical evidence. Sleep Med Rev. 2015;22:23–36. 25454674 10.1016/j.smrv.2014.10.001 PMC4400203

[21] Brand S, Kirov R. Sleep and its importance in adolescence and in common adolescent somatic and psychiatric conditions. Int J Gen Med. 2011. 10.2147/IJGM.S11557 21731894 10.2147/IJGM.S11557 PMC3119585

[22] Matricciani L, Blunden S, Rigney G, Williams MT, Olds TS. Children’s sleep needs: is there sufficient evidence to recommend optimal sleep for children? Sleep. 2013;36(4):527–34. 23564999 10.5665/sleep.2538 PMC3612266

[23] Altena E, Baglioni C, Espie CA, Ellis J, Gavriloff D, Holzinger B, et al. Dealing with sleep problems during home confinement due to the COVID-19 outbreak: practical recommendations from a task force of the European CBT-I academy. J Sleep Res. 2020;29(4):e13052. 32246787 10.1111/jsr.13052

[24] Mindell JA, Li AM, Sadeh A, Kwon R, Goh DY. Bedtime routines for young children: a dose-dependent association with sleep outcomes. Sleep. 2015;38(5):717–22. 25325483 10.5665/sleep.4662 PMC4402657

[25] Mindell JA, Williamson AA. Benefits of a bedtime routine in young children: sleep, development, and beyond. Sleep Med Rev. 2018;40:93–108. 29195725 10.1016/j.smrv.2017.10.007 PMC6587181

[26] Mindell JA, Telofski LS, Wiegand B, Kurtz ES. A nightly bedtime routine: impact on sleep in young children and maternal mood. Sleep. 2009;32(5):599–606. 19480226 10.1093/sleep/32.5.599 PMC2675894

[27] Mindell JA, Meltzer LJ, Carskadon MA, Chervin RD. Developmental aspects of sleep hygiene: findings from the 2004 National Sleep Foundation sleep in America poll. Sleep Med. 2009;10(7):771–9. 19285450 10.1016/j.sleep.2008.07.016

[28] Zisberg A, Gur-Yaish N, Shochat T. Contribution of routine to sleep quality in community elderly. Sleep. 2010;33(4):509–14. 20394320 10.1093/sleep/33.4.509 PMC2849790

[29] Taub JM. Behavioral and psychophysiological correlates of irregularity in chronic sleep routines. Biol Psychol. 1978;7(1–2):37–53. 218642 10.1016/0301-0511(78)90041-8

[30] Wainberg M, Jones SE, Beaupre LM, Hill SL, Felsky D, Rivas MA, et al. Association of accelerometer-derived sleep measures with lifetime psychiatric diagnoses: a cross-sectional study of 89,205 participants from the UK Biobank. PLoS Med. 2021;18(10):e1003782. 34637446 10.1371/journal.pmed.1003782 PMC8509859

[31] Becker SP, Sidol CA, Van Dyk TR, Epstein JN, Beebe DW. Intraindividual variability of sleep/wake patterns in relation to child and adolescent functioning: a systematic review. Sleep Med Rev. 2017;34:94–121. 27818086 10.1016/j.smrv.2016.07.004 PMC5253125

[32] Slavish DC, Taylor DJ, Lichstein KL. Intraindividual variability in sleep and comorbid medical and mental health conditions. Sleep. 2019;42(6):zsz052. 30843059 10.1093/sleep/zsz052 PMC6559172

[33] Choi KW, Stein MB, Nishimi KM, Ge T, Coleman JR, Chen CY, et al. An exposure-wide and Mendelian randomization approach to identifying modifiable factors for the prevention of depression. Am J Psychiatry. 2020;177(10):944–54. 32791893 10.1176/appi.ajp.2020.19111158 PMC9361193

[34] Zheng NS, Annis J, Master H, Han L, Gleichauf K, Ching JH, et al. Sleep patterns and risk of chronic disease as measured by long-term monitoring with commercial wearable devices in the All of Us research program. Nat Med. 2024;30(9). 10.1038/s41591-024-03155-8. 10.1038/s41591-024-03155-8 PMC11405268 39030265

[35] Messman BA, Wiley JF, Feldman E, Dietch JR, Taylor DJ, Slavish DC. Irregular sleep is linked to poorer mental health: a pooled analysis of eight studies. Sleep Health. 2024;10(4):493–9. 38704353 10.1016/j.sleh.2024.03.004 PMC12036700

[36] Windred DP, Burns AC, Lane JM, Saxena R, Rutter MK, Cain SW, et al. Sleep regularity is a stronger predictor of mortality risk than sleep duration: a prospective cohort study. Sleep. 2024;47(1):zsad253. 37738616 10.1093/sleep/zsad253 PMC10782501

[37] Wyse CA, Biello SM, Gill JM. The bright-nights and dim-days of the urban photoperiod: implications for circadian rhythmicity, metabolism and obesity. Ann Med. 2014;46(5):253–63. 24901354 10.3109/07853890.2014.913422

[38] West AC, Bechtold DA. The cost of circadian desynchrony: evidence, insights and open questions. Bioessays. 2015;37(7):777–88. 26010005 10.1002/bies.201400173 PMC4973832

[39] Doherty A, Jackson D, Hammerla N, Plötz T, Olivier P, Granat MH, et al. Large scale population assessment of physical activity using wrist worn accelerometers: the UK biobank study. PLoS ONE. 2017;12(2):e0169649. 28146576 10.1371/journal.pone.0169649 PMC5287488

[40] UKBiobank. Data providers and dates of data availability. 2023. https://biobank.ndph.ox.ac.uk/ukb/exinfo.cgi?src=Data_providers_and_dates. Accessed 31 Aug 2023.

[41] Wood SN. Generalized Additive Models: An Introduction with R. 2nd ed. Chapman and Hall/CRC; 2017.

[42] Wood SN. Thin-plate regression splines. J R Stat Soc B. 2003;65(1):95–114.

[43] Mamun A, Paul S. Model selection in generalized linear models. Symmetry. 2023;15(10):1905.

[44] Moebus M, Gashi S, Hilty M, Oldrati P, Holz C. Meaningful digital biomarkers derived from wearable sensors to predict daily fatigue in multiple sclerosis patients and healthy controls. iScience. 2024. 10.1016/j.isci.2024.108965 10.1016/j.isci.2024.108965 PMC10867654 38362266

[45] Royston P, Altman DG. External validation of a Cox prognostic model: principles and methods. BMC Med Res Methodol. 2013;13:1–15. 23496923 10.1186/1471-2288-13-33 PMC3667097

[46] McLernon DJ, Giardiello D, Van Calster B, Wynants L, van Geloven N, van Smeden M, et al. Assessing performance and clinical usefulness in prediction models with survival outcomes: practical guidance for Cox proportional hazards models. Ann Intern Med. 2023;176(1):105–14. 36571841 10.7326/M22-0844

[47] Moons KG, Altman DG, Reitsma JB, Ioannidis JP, Macaskill P, Steyerberg EW, et al. Transparent Reporting of a multivariable prediction model for Individual Prognosis or Diagnosis (TRIPOD): explanation and elaboration. Ann Intern Med. 2015;162(1):W1–73. 25560730 10.7326/M14-0698

[48] Harrell FE, Califf RM, Pryor DB, Lee KL, Rosati RA. Evaluating the yield of medical tests. JAMA. 1982;247(18):2543–6. 7069920

[49] Uno H, Cai T, Pencina MJ, D’Agostino RB, Wei LJ. On the C-statistics for evaluating overall adequacy of risk prediction procedures with censored survival data. Stat Med. 2011;30(10):1105–17. 21484848 10.1002/sim.4154 PMC3079915

[50] VanderWeele TJ, Ding P. Sensitivity analysis in observational research: introducing the E-value. Ann Intern Med. 2017;167(4):268–74. 28693043 10.7326/M16-2607

[51] Handy A, Mangal R, Stead TS, Coffee RL Jr., Ganti L. Prevalence and impact of diagnosed and undiagnosed depression in the United States. Cureus. 2022. 10.7759/cureus.28011. 10.7759/cureus.28011 PMC9470500 36134073

[52] Harris M, Glozier N, Ratnavadivel R, Grunstein RR. Obstructive sleep apnea and depression. Sleep Med Rev. 2009;13(6):437–44. 19596599 10.1016/j.smrv.2009.04.001

[53] Klein JP, Moeschberger ML, et al. Survival analysis: techniques for censored and truncated data, vol. 1230. Springer; 2003.

[54] Walmsley R, Chan S, Smith-Byrne K, Ramakrishnan R, Woodward M, Rahimi K, et al. Reallocation of time between device-measured movement behaviours and risk of incident cardiovascular disease. Br J Sports Med. 2022;56(18):1008–17. 10.1136/bjsports-2021-104050. 10.1136/bjsports-2021-104050 PMC9484395 34489241

[55] Hirshkowitz M, Whiton K, Albert SM, Alessi C, Bruni O, DonCarlos L, et al. National Sleep Foundation’s updated sleep duration recommendations. Sleep Health. 2015;1(4):233–43. 29073398 10.1016/j.sleh.2015.10.004

[56] Grandner MA, Patel NP, Gehrman PR, Xie D, Sha D, Weaver T, et al. Who gets the best sleep? Ethnic and socioeconomic factors related to sleep complaints. Sleep Med. 2010;11(5):470–8. 20388566 10.1016/j.sleep.2009.10.006 PMC2861987

[57] Abel T. Cultural capital and social inequality in health. J Epidemiol Community Health. 2008;62(7):e13–e13. 18572429 10.1136/jech.2007.066159

[58] Goldman N. Social inequalities in health: disentangling the underlying mechanisms. Ann N Y Acad Sci. 2001;954(1):118–39. 11797854

[59] Mackenbach JP, Meerding WJ, Kunst AE. Economic costs of health inequalities in the European Union. J Epidemiol Community Health. 2011;65(5):412–9. 21172799 10.1136/jech.2010.112680

[60] Fryers T, Melzer D, Jenkins R, Brugha T. The distribution of the common mental disorders: social inequalities in Europe. Clin Pract Epidemiol Ment Health. 2005;1(1):1–12. 16143042 10.1186/1745-0179-1-14 PMC1242241

[61] Cheng P, Walch O, Huang Y, Mayer C, Sagong C, Cuamatzi Castelan A, et al. Predicting circadian misalignment with wearable technology: validation of wrist-worn actigraphy and photometry in night shift workers. Sleep. 2021;44(2):zsaa180. 32918087 10.1093/sleep/zsaa180 PMC8240654

[62] Pandi-Perumal SR, Monti JM, Burman D, Karthikeyan R, BaHammam AS, Spence DW, et al. Clarifying the role of sleep in depression: a narrative review. Psychiatry Res. 2020;291:113239. 32593854 10.1016/j.psychres.2020.113239

[63] Benca RM, Peterson MJ. Insomnia and depression. Sleep Med. 2008;9:S3-9. 18929317 10.1016/S1389-9457(08)70010-8

[64] Lopez R, Barateau L, Evangelista E, Dauvilliers Y. Depression and hypersomnia: a complex association. Sleep Med Clin. 2017;12(3):395–405. 28778237 10.1016/j.jsmc.2017.03.016

[65] Schmid M, Wright MN, Ziegler A. On the use of Harrell’s c for clinical risk prediction via random survival forests. Expert Syst Appl. 2016;63:450–9.

[66] Van Belle V, Pelckmans K, Van Huffel S, Suykens JA. Improved performance on high-dimensional survival data by application of Survival-SVM. Bioinformatics. 2011;27(1):87–94. 21062763 10.1093/bioinformatics/btq617

[67] Swanson JM, The UK. Biobank and selection bias. Lancet. 2012;380(9837):110. 22794246 10.1016/S0140-6736(12)61179-9

[68] Fry A, Littlejohns TJ, Sudlow C, Doherty N, Adamska L, Sprosen T, et al. Comparison of sociodemographic and health-related characteristics of UK Biobank participants with those of the general population. Am J Epidemiol. 2017;186(9):1026–34. 28641372 10.1093/aje/kwx246 PMC5860371

[69] Lublóy Á, Keresztúri JL, Németh A, Mihalicza P. Exploring factors of diagnostic delay for patients with bipolar disorder: a population-based cohort study. BMC Psychiatr. 2020;20:1–17. 10.1186/s12888-020-2483-y PMC7031950 32075625

[70] Chee MW, Baumert M, Scott H, Cellini N, Goldstein C, Baron K, et al. World sleep society recommendations for the use of wearable consumer health trackers that monitor sleep. Sleep Med. 2025;131:106506. 10.1016/j.sleep.2025.106506. 40300398 10.1016/j.sleep.2025.106506

[71] Miller DJ, Roach GD, Lastella M, Scanlan AT, Bellenger CR, Halson SL, et al. A validation study of a commercial wearable device to automatically detect and estimate sleep. Biosensors. 2021;11(6):185. 34201016 10.3390/bios11060185 PMC8226553

[72] Sudlow C, Gallacher J, Allen N, Beral V, Burton P, Danesh J, et al. UK biobank: an open access resource for identifying the causes of a wide range of complex diseases of middle and old age. PLoS Med. 2015;12(3):e1001779. 25826379 10.1371/journal.pmed.1001779 PMC4380465

