# Supplementary material for: Beyond the hours slept: inconsistent sleep routines threaten mental health in 100,000 UK Biobank participants
Source: BMC Public Health. 2025 Nov 17;25:4009. doi: 10.1186/s12889-025-24794-7 (PMC12625614; doi:10.1186/s12889-025-24794-7)
Supplement: Supplementary file 1 — Supplementary material. Fig. A1: Derivation of the number of routine sleep hours for an exemplary participant. Fig. A2: Stratification of the 2D sleep routine effect. Fig. A3: Effects of the Control Variables. Fig. A4: Kaplan Meier survival curves. [file 12889_2025_24794_MOESM1_ESM.pdf]

Appendix: Extended Data

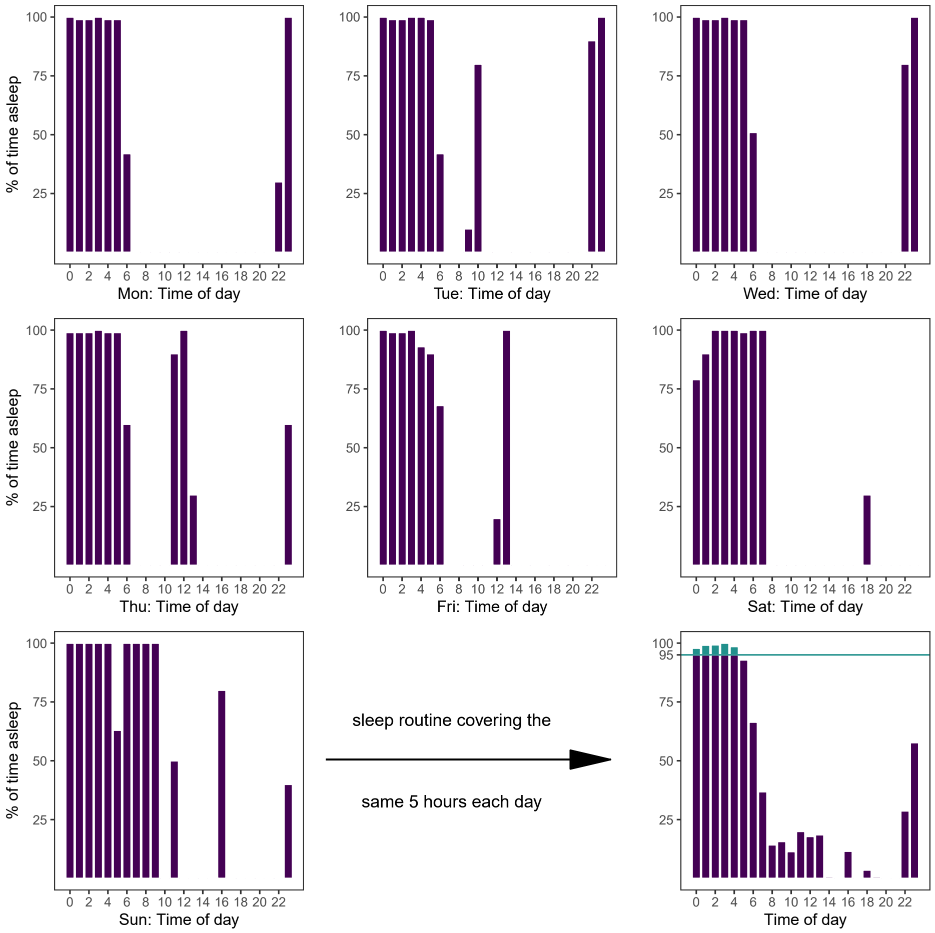

Fig. A1: Derivation of the number of routine sleep hours for an exemplary participant. We divide the day into 24 fixed 1-hour windows and average the proportion of time participants were asleep for during each 1-hour window across the entire week. We count each of the 24 1-hour windows as a routine sleep hour if participants were asleep for more than 95% of the time during that window over the entire week. The plot in the bottom right corner is the average of the 7 daily plots. The 1-hour windows that cross the 95% threshold are counted as a routine sleep hour are marked in green at the top.

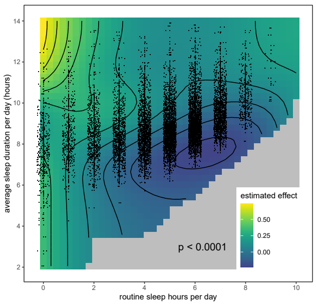

(a) all participants

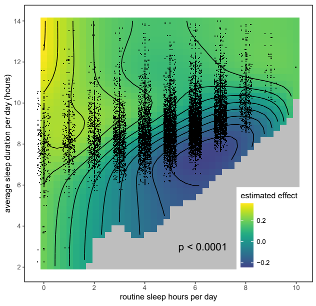

(b) female participants

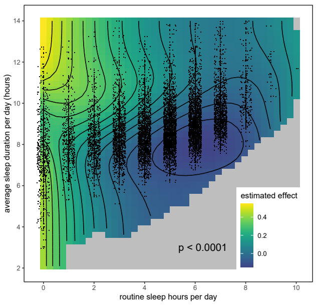

(c) male participants

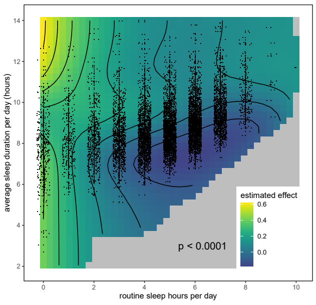

(d) younger participants

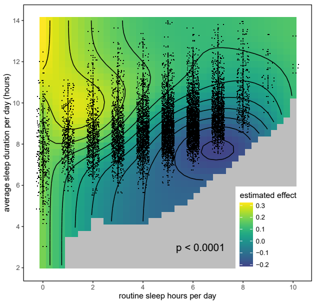

(e) older participants

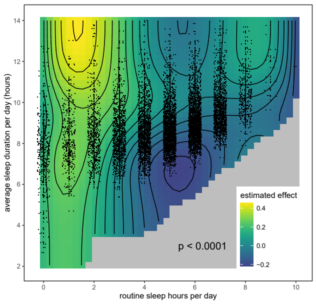

(f) excluding first two years

Fig. A2: Evaluation of the 2D sleep routine effect when the model is fitted for all participants at once (same as Figure 2a), during age- and gender-based stratification, and when any diagnosis occurring during the first two years after the sleep behavior study is ignored (Figure A2f). In regions with sufficient data, the plots show a high level of agreement, and the shape of the 2D sleep routine effect remains almost constant across the different subgroups highlighting the importance of the interplay of sleep routine and duration. Figure A2b shows the 2D sleep routine effect when the model is fitted only for female participants, Figure A2c only for male participants, Figure A2d only for the younger half of participants (younger than 63.7 years at the time of the sleep behavior study), and Figure A2e only for the older half of participants.

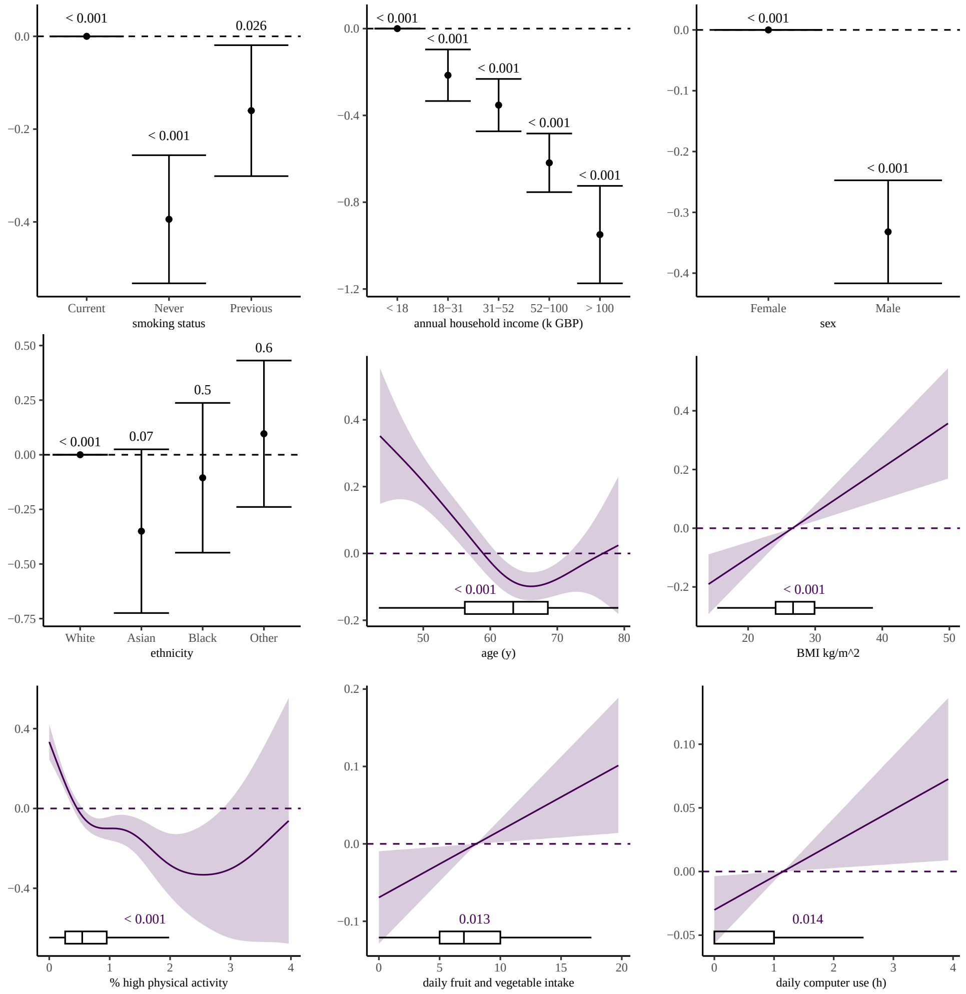

Fig. A3: Effect plots on the hazard ratio of developing mental disorders for the control variables of the multivariate generalized additive Cox proportional hazard model. To rule out hidden confounders, we controlled for a broad array of variables suggested as predictors for mental disorders by related works.

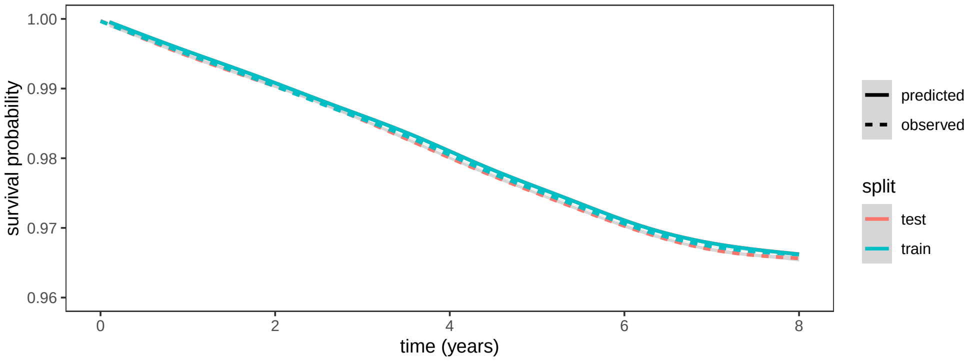

Fig. A4: High agreement between Kaplan Meier survival curves and predicted survival curves across a training split (90% of the data) and a completely unseen test split (10% of the data) for our multivariate generalized additive Cox proportional hazard model. The curves correspond to Harrel's and Uno's C-statistics with a value of 0.634 on the train splits and 0.625 on the test splits demonstrating robustness towards unseen data.
